# Impact of dactylitis and enthesitis resolution on disease control in guselkumab-treated psoriatic arthritis patients with TNFi-IR: COSMOS *post hoc* analysis

**DOI:** 10.1093/rheumatology/keaf490

**Published:** 2025-09-14

**Authors:** Helena Marzo-Ortega, Iain B McInnes, Mohamed Sharaf, Alen Zabotti, Emmanouil Rampakakis, Dennis McGonagle, Ahmed Abogamal, Pascal Richette, Georg Schett

**Affiliations:** NIHR Leeds Biomedical Research Centre, The Leeds Teaching Hospitals NHS Trust and Leeds Institute of Rheumatic and Musculoskeletal Medicine, University of Leeds, Leeds, UK; MVLS College Office, University of Glasgow, Glasgow, UK; Johnson & Johnson, Middle East TZ LLC, Dubai, UAE; Rheumatology Institute, Azienda Sanitaria Universitaria del Friuli Centrale, Udine, Italy; Department of Pediatrics, McGill University, Montreal, QC, Canada; JSS Medical Research Inc., Scientific Affairs, Montreal, QC, Canada; NIHR Leeds Biomedical Research Centre, The Leeds Teaching Hospitals NHS Trust and Leeds Institute of Rheumatic and Musculoskeletal Medicine, University of Leeds, Leeds, UK; Department of Rheumatology, Al Zahra Hospital, Dubai, UAE; Al-Azhar Faculty of Medicine, Al-Azhar University, Cairo, Egypt; Rheumatology Department, Lariboisière Hospital, Paris Cité University, AP-HP, Paris, France; Department of Medicine 3—Rheumatology and Immunology, Friedrich-Alexander-Universität Erlangen-Nürnberg and Universitätsklinikum Erlangen, Erlangen, Germany

**Keywords:** guselkumab, psoriatic arthritis, dactylitis, enthesitis, resolution, clinical outcomes, tumour necrosis factor inhibitor, COSMOS

## Abstract

**Objective:**

To evaluate guselkumab efficacy on dactylitis resolution (DR) and enthesitis resolution (ER), and their impact on subsequent disease control, in patients with active psoriatic arthritis (PsA) and prior inadequate response to tumour necrosis factor inhibitors (TNFi-IR).

**Methods:**

In the Phase IIIb COSMOS trial, 285 adults with TNFi-IR PsA were randomized (2:1) to receive guselkumab 100 mg or placebo at Week (W)0, W4, then every 8 weeks until W44. The Dactylitis Severity Score (DSS) and Leeds Enthesitis Index (LEI) assessed dactylitis and enthesitis, respectively. This *post hoc* analysis evaluated associations between W24 DR or ER and W48 achievement of stringent disease control measures using logistic regression.

**Results:**

At baseline, 103/285 (36.1%) patients had dactylitis (DSS ≥ 1) and 190/285 (66.7%) had enthesitis (LEI ≥ 1). Patients with dactylitis were more likely to have enthesitis, more joint (SJC/DAPSA) and skin involvement, higher PGA score and lower BMI vs those without dactylitis. Patients with enthesitis were more likely to be female, and have dactylitis, more joints affected (SJC/TJC/DAPSA) and worse physical functioning (HAQ-DI/SF-36 PCS) vs those without enthesitis. Greater proportions of guselkumab- vs placebo-treated patients achieved DR/ER (W24: 44.8%/39.7% vs 25.0%/18.8%); rates increased through W48 among guselkumab-randomized patients (67.2%/55.6%). W24 resolution was associated with W48 achievement of stringent measures, including ACR50/70, DAPSA LDA/remission, PASI100, PASDAS LDA/VLDA and MDA/VLDA (odds ratios: DR, 3.28–13.38; ER, 2.88–6.09).

**Conclusion:**

Guselkumab treatment resulted in high DR/ER rates through W48 in TNFi-IR PsA patients. W24 DR/ER was associated with W48 disease control, providing valuable insights for clinical decision-making based on W24 treatment responses.

Rheumatology key messages

- PsA patients with dactylitis or enthesitis experience more severe disease and poorer outcomes than those without.
- Guselkumab-treated patients achieved high dactylitis and enthesitis resolution response rates through W48.
- Dactylitis and enthesitis resolution at W24 were associated with achievement of stringent measures of disease control at W48.

## Introduction

Psoriatic arthritis (PsA) is a complex inflammatory, musculoskeletal and cutaneous disease with heterogeneous clinical presentation and variable course [1, 2]. The musculoskeletal manifestations include dactylitis and enthesitis, peripheral arthritis and axial disease [3]. The ClASsification criteria for Psoriatic ARthritis (CASPAR) signs and symptoms also include evidence of psoriasis (current or personal/family history of psoriasis), psoriatic nail lesions, rheumatoid factor negativity and juxta-articular new bone formation [3].

Dactylitis is diffuse swelling of a whole digit, resulting in ‘sausage shaped’ fingers or toes, alongside inflammation in the joints, soft tissues and tendon sheaths. Dactylitis affects between 16% and 50% of patients with PsA [4, 5], and its presence typically indicates a more severe disease phenotype. Patients with dactylitis have a higher burden of disease [6, 7] and more radiographic joint damage [8] than those without it. Magnetic resonance imaging (MRI) studies have demonstrated that dactylitis inherently involves enthesitis, manifesting as abnormally thickened flexor tendon pulleys and entheseal changes [9]. Enthesitis is characterized by inflammation of the tendon or ligament insertion sites to the bone, and affects between 35% and 54% of patients with PsA [5, 10]. Affected patients are also more likely to have more active disease and greater pain than patients without enthesitis [11]. Therefore, therapies that treat or prevent the development of these manifestations of PsA are of importance [8].

Interleukin (IL)-23, secreted from myeloid cells in the enthesis [12], is a key driver in the pathogenesis of both dactylitis and enthesitis [13–15]. Guselkumab is a monoclonal antibody that targets the p19 subunit of IL-23 and is approved for the treatment of active PsA, as well as for moderate-to-severe psoriasis and moderately to severely active ulcerative colitis [16–18]. The Phase III DISCOVER-1 [19] and DISCOVER-2 [20] clinical trials evaluated guselkumab efficacy, compared with placebo, in patients with active PsA. Most patients (89.5%) were biologic-naïve. Pooled analyses of these two trials determined that guselkumab 100 mg dosing every 4 weeks (Q4W) or every 8 weeks (Q8W) was significantly superior to placebo in resolving pre-existing dactylitis (63.5% or 59.4% vs 42.2%, respectively) or enthesitis (44.9% or 49.6% vs 29.4%, respectively) at Week (W)24, and resolution rates increased further from W24 through W52 [4, 10].

Patients with PsA and a history of inadequate response to tumour necrosis factor inhibitors (TNFi) can be less responsive to, and have lower treatment persistence with, subsequent TNFi therapies or other biologic therapies [21, 22]. The Phase IIIb COSMOS trial was conducted to evaluate the effect of guselkumab Q8W, compared with placebo, in patients with PsA and an inadequate response to one or two TNFi (TNFi-IR) [23]. Among patients with dactylitis or enthesitis at baseline, numerically higher proportions of patients randomized to guselkumab, compared with patients randomized to placebo, achieved dactylitis resolution (44.8% vs 25.0%) and enthesitis resolution (39.7% vs 18.8%) at W24 [23].

In these *post hoc* analyses of COSMOS, we further evaluate the effect of guselkumab, compared with placebo, in resolving pre-existing dactylitis or enthesitis in patients with TNFi-IR through to W24, and from W24 to W48 in the guselkumab-randomized group. We also examine the impact of dactylitis or enthesitis resolution on future achievement of stringent measures of disease control at W48.

## Methods

### Patients

Eligible adults had a diagnosis of PsA, fulfilling the CASPAR classification criteria at screening, with active joint (tender joint count [TJC] and swollen joint count [SJC] both ≥3) and skin involvement (≥1 psoriatic plaque of ≥2 cm) or documented history of plaque psoriasis or current nail psoriasis, and had TNFi-IR, defined as a demonstrated lack of benefit or intolerance to one or two TNFi. Patients could continue stable baseline use of methotrexate (MTX), sulfasalazine, hydroxychloroquine or leflunomide, oral corticosteroids, and non-steroidal anti-inflammatory drugs/other analgesics. Targeted synthetic disease-modifying antirheumatic drugs (DMARDs) were prohibited before and during study participation. Patients with active tuberculosis were excluded; patients with latent tuberculosis received appropriate prophylaxis.

### Trial design

COSMOS was a Phase IIIb, randomized, double-blind, placebo-controlled clinical trial (NCT03796858) conducted across 84 European sites between March 2019 and November 2020 [23]. The overall COSMOS trial design has been described previously [23]. In summary, adults with TNFi-IR PsA were randomized 2:1 to receive guselkumab 100 mg at W0 and W4, then Q8W through W44 (final assessment at W48), or to placebo at W0, W4, W12 and W20 with crossover to receive guselkumab 100 mg at W24, W28, W36 and W44. Patients with <5% improvement in both TJC and SJC between baseline and W16 qualified for early escape; those receiving guselkumab continued their treatment (including a placebo dose at W16 to maintain blinding), while those in the placebo group received guselkumab at W16 and W20, then Q8W through to W44. Patients who met early escape criteria could initiate or increase the dose of one permitted concomitant medication, up to the maximum dose allowed by protocol, at the physician’s discretion.

COSMOS was conducted in accordance with the Declaration of Helsinki and Good Clinical Practice guidelines, and all participants provided written informed consent. The trial protocol was approved by the governing ethical body at each site.

### Assessments

Efficacy assessments and outcome measures in COSMOS have been reported previously [23]. Dactylitis was assessed using the Dactylitis Severity Score (DSS), in which independent joint assessors evaluated the response to squeezing, with moderate pressure, all 20 digits of each patient, categorizing each digit as not tender (0), tender (1), tender and winces (2) or tender and withdraws (3; DSS score range: 0–60) [24]. Enthesitis was assessed using the Leeds Enthesitis Index (LEI), in which the absence (0) or presence (1) of painful entheses among the left and right lateral epicondyles of the humerus, left and right medial femoral condyles, and left and right Achilles tendon insertions was documented (LEI score range: 0–6) [25]. The current *post hoc* analyses evaluated dactylitis and enthesitis resolution rates through to W48 (DSS = 0 and LEI = 0, respectively), the proportion of patients with new-onset dactylitis or enthesitis, and the association between dactylitis or enthesitis resolution at W24 and the achievement of stringent measures of disease control at W48. Stringent measures of disease control included: (1) ≥50%/70% improvement in American College of Rheumatology (ACR) criteria (ACR 50/70 response) [26]; (2) Disease Activity in Psoriatic Arthritis (DAPSA) score ≤14, indicative of low disease activity (LDA), or DAPSA score ≤4, indicative of remission [27]; (3) 100% improvement in the Psoriasis Area and Severity Index (PASI 100 response) [28]; (4) Psoriatic Arthritis Disease Activity Score (PASDAS) ≤3.2, indicative of LDA, or PASDAS score ≤1.9, indicative of very low disease activity (VLDA) [27]; and (5) minimal disease activity (MDA) or VLDA, defined as achievement of five or seven of the following criteria, respectively: TJC ≤1, SJC ≤1, PASI ≤1, patient pain on a visual analogue scale [VAS; 0–10 cm] ≤1.5 cm, Patient’s Global Assessment of disease activity [0–10 cm VAS] ≤2.0 cm, Health Assessment Questionnaire—Disability Index [HAQ-DI] ≤0.5, and LEI ≤1 [27, 29].

### Statistical analyses

Baseline characteristics were evaluated in cohorts of patients with or without dactylitis or enthesitis, and per treatment group; the independent-samples *t* test was used to compare continuous variables between the groups, and the chi-squared test was used to compare categorical variables between the groups.

As previously published [23], efficacy analyses were conducted using the full analysis set from COSMOS, which included all randomized patients who received ≥1 dose of study agent. Dactylitis and enthesitis analyses were conducted on patients in the full analysis set who had a DSS or LEI of ≥1 at baseline, respectively. Patients with missing data and those who met treatment failure criteria through W24 (i.e. discontinued study agent and/or study participation for any reason, initiated or increased the dose of allowed conventional synthetic DMARDs [csDMARDs] or oral corticosteroids for PsA, initiated protocol-prohibited medications/therapies for PsA, or met early escape criteria) were considered non-responders (i.e. non-responder imputation [NRI]/composite analysis). Analyses beyond W24 did not account for treatment failure (i.e. NRI analysis only), and only patients randomized to guselkumab were included. Dactylitis and enthesitis resolution rates for guselkumab vs placebo were compared using the Cochran–Mantel–Haenszel Test, stratified by baseline use of csDMARD (yes/no) and prior exposure to TNFi (1 vs 2) [23].

For *post hoc* analyses of new-onset dactylitis or enthesitis, as-observed data were used. Associations between dactylitis or enthesitis resolution at W24 and achievement of stringent measures of disease control at W48, including odds ratios (ORs), 95% Wald confidence intervals (95% CIs) and *P*-values, were determined using a logistic regression model. The analyses were not powered to detect statistically significant differences; reported *P*-values are nominal.

## Results

### Patients and baseline characteristics

#### Overall population

A total of 285 patients with TNFi-IR PsA underwent randomization, of whom 189 were assigned to guselkumab and 96 to placebo. At W16, 39 (20.6%) patients in the guselkumab arm and 45 (46.9%) patients in the placebo arm qualified for early escape. By W24, 15 (7.9%) and eight (8.3%) patients had discontinued treatment in the guselkumab and placebo groups, respectively. Overall, 51 (53.1%) patients initially randomized to placebo switched to guselkumab at W24 as planned. At W44, 167 (88.4%) patients initially randomized to guselkumab and 83 (86.4%) patients initially randomized to placebo had completed the study treatment.

Baseline demographics and disease characteristics were generally similar between the guselkumab and placebo groups and have been published previously [23]. All 285 patients had received one prior TNFi, and 33 (11.6%) had received two; 156 (54.7%) patients were receiving concomitant MTX.

#### Patients with dactylitis

Of the total population, 103/285 (36.1%) had dactylitis (DSS ≥1: guselkumab, 67/189 [35.4%]; placebo, 36/96 [37.5%]) at baseline. The mean DSS score among those affected was 6.9 (standard deviation [S.D.] 7.1; Table 1) and the mean number of digits affected (out of 20) was 3.9 (S.D. 3.5). Most baseline characteristics were similar between the treatment groups for patients with dactylitis, though patients in the guselkumab group were more likely (*P* < 0.05) than those in the placebo group to have higher DAPSA (52.0 vs 42.7), patient pain (7.0 vs 5.9), HAQ-DI (1.4 vs 1.1), Patient’s Global Assessment of arthritis (PtGA; 7.0 vs 6.2) and Physician’s Global Assessment (PGA; 7.2 vs 6.5) scores (Supplementary Table S1). Conversely, the placebo group had higher mean 36-item short-form health survey physical component summary (SF-36 PCS) scores than the guselkumab group (36.4 vs 32.9).

**Table 1.** Baseline demographics and disease characteristics

Characteristic/measure	Dactylitis at baseline		Enthesitis at baseline	
	Yes (*n* = 103)	No (*n* = 180)	Yes (*n* = 190)	No (*n* = 93)
Demographics				
Age, years	48.3 (11.8)	49.6 (12.4)	49.9 (11.7)	47.7 (13.1)
<65 years, *n* (%)	93 (90.3)	163 (90.6)	171 (90.0)	85 (91.4)
≥65 years, *n* (%)	10 (9.7)	17 (9.4)	19 (10.0)	8 (8.6)
Sex				
Male, *n* (%)	51 (49.5)	86 (47.8)	82 (43.2)^a^	55 (59.1)^a^
Female, *n* (%)	52 (50.5)	94 (52.2)	108 (56.8)	38 (40.9)
Weight, kg	83.9 (20.1)	87.6 (19.4)	86.4 (20.8)	86.1 (17.4)
BMI, kg/m^2^	28.5 (5.9)^a^	30.1 (6.6)^a^^,^^b^	29.8 (6.4)	29.1 (6.2)^c^
Musculoskeletal manifestations				
SJC, 0–66	12.3 (8.1)^a^	8.5 (4.7)^a^	10.8 (7.2)^a^	7.9 (3.8)^a^
TJC, 0–68	22.0 (12.9)	19.1 (12.1)	23.1 (13.2)^a^	13.9 (8.0)^a^
DAPSA score	48.7 (21.0)^a^^,^^d^	41.2 (16.8)^a^	48.0 (19.9)^a^	35.5 (12.5)^a^^,^^c^
Periarticular manifestations				
DSS ≥1, *n* (%)	103 (100.0)^a^	0 (0.0)^a^	78 (41.1)^a^	25 (26.9)^a^
DSS score, 1–60	6.9 (7.1)	–	7.9 (7.9)	4.0 (2.1)
LEI ≥1, *n* (%)	78 (75.7)^a^	112 (62.2)^a^	190 (100.0)^a^	0 (0.0)^a^
LEI score, 1–6	3.1 (1.4)^a^	2.6 (1.5)^a^	2.8 (1.5)	–
Skin measures				
PASI, 0–72	14.6 (12.7)^a^^,^^d^	8.7 (9.6)^a^	11.3 (12.3)^e^	9.9 (8.4)
Psoriatic BSA, %	23.4 (23.6)^a^	12.3 (17.1)^a^	17.1 (22.4)	14.7 (15.5)
IGA <2, *n* (%)	20 (19.4)	49 (27.2)	48 (25.3)	21 (22.6)
IGA ≥2, *n* (%)	83 (80.6)	131 (72.8)	142 (74.7)	72 (77.4)
Patient-reported measures				
Patient pain, 0–10 cm VAS	6.6 (1.7)	6.2 (2.0)	6.4 (1.7)	6.1 (2.1)
HAQ-DI, 0–3	1.3 (0.6)	1.3 (0.6)	1.4 (0.6)^a^	1.1 (0.6)^a^
PtGA, 0–10 cm VAS	6.8 (1.7)	6.6 (1.8)	6.7 (1.7)	6.5 (1.9)
FACIT-F score, 0–52	30.6 (11.1)	28.4 (10.9)	28.6 (11.4)	30.4 (10.2)
SF-36 PCS score, 0–100	34.1 (6.8)	32.9 (7.5)	32.2 (7.2)^a^	35.5 (6.9)^a^
SF-36 MCS score, 0–100	47.5 (12.1)	46.3 (11.7)	47.4 (12.3)	45.3 (10.8)
Physician-reported measures				
PGA, 0–10 cm VAS	7.0 (1.4)^a^	6.6 (1.7)^a^	6.8 (1.6)	6.5 (1.7)
PASDAS	7.0 (0.9)^d^	6.0 (0.8)	6.6 (0.9)	5.8 (0.9)^c^
CRP, mg/dL	1.2 (2.3)^d^	1.2 (2.1)	1.1 (2.1)	1.4 (2.3)^c^
Prior/ongoing therapies				
One prior TNFi, *n* (%)	91 (88.3)	159 (88.3)	167 (87.9)	83 (89.2)
Two prior TNFi, *n* (%)	12 (11.7)	21 (11.7)	23 (12.1)	10 (10.8)
Reason for prior TNFi discontinuation				
Efficacy, *n* (%)	83 (80.6)	148 (82.2)	155 (81.6)	76 (81.7)
Safety, *n* (%)	13 (12.6)	29 (16.1)	27 (14.2)	15 (16.1)
Other, *n* (%)	7 (6.8)	3 (1.7)	8 (4.2)	2 (2.2)
MTX use at baseline, *n* (%)	57 (55.3)	97 (53.9)	102 (53.7)	52 (55.9)

Data are mean (S.D.) unless otherwise stated.

a Yes vs no *P* < 0.05.

b *n* = 179.

c *n* = 92.

d *n* = 102.

e *n* = 189.

BMI, body mass index; BSA, body surface area; CRP, C-reactive protein; DAPSA, Disease Activity in Psoriatic Arthritis; DSS, Dactylitis Severity Score; FACIT-F, Functional Assessment of Chronic Illness Therapy—Fatigue; HAQ-DI, Heath Assessment Questionnaire—Disability Index; IGA, Investigator’s Global Assessment; LEI, Leeds Enthesitis Index; MTX, methotrexate; PASDAS, Psoriatic Arthritis Disease Activity Score; PASI, Psoriasis Area and Severity Index; PGA, Physician’s Global Assessment; PtGA, Patient’s Global Assessment of arthritis; S.D., standard deviation; SF-36 MCS, 36-item short-form health survey mental component summary; SF-36 PCS, 36-item short-form health survey physical component summary; SJC, swollen joint count; TJC, tender joint count; TNFi, tumour necrosis factor inhibitor; VAS, visual analogue scale.

Patients with dactylitis were more likely (*P* < 0.05) than those without dactylitis to have enthesitis (75.7% vs 62.2%), and a higher mean SJC (12.3 vs 8.5) and DAPSA score (48.7 vs 41.2). Furthermore, patients with dactylitis had greater skin involvement (PASI: 14.6 vs 8.7; psoriatic body surface area [BSA]: 23.4% vs 12.3%) and a higher PGA (7.0 vs 6.6) score. Of note, the dactylitis group had a lower mean BMI (28.5 vs 30.1 kg/m^2^; Table 1).

#### Patients with enthesitis

Overall, 190/285 (66.7%) patients had enthesitis (LEI ≥1: guselkumab, 126/189 [66.7%]; placebo, 64/96 [66.7%]), with a mean LEI score of 2.8 (S.D. 1.5) at baseline (Table 1). Among these patients, most baseline characteristics were similar between the treatment groups, though patients in the guselkumab group were more likely (*P* < 0.05) than those in the placebo group to have higher DAPSA (50.2 vs 43.5) and PGA (7.0 vs 6.5) scores (Supplementary Table S1). Conversely, the placebo group had a higher mean weight (93.3 vs 82.8 kg) and BMI (31.5 vs 28.9 kg/m^2^) than the guselkumab group.

Patients with enthesitis were more likely (*P* < 0.05) than patients without enthesitis to have dactylitis (41.1% vs 26.9%), be female (56.8% vs 40.9%) and to have higher mean SJC (10.8 vs 7.9), TJC (23.1 vs 13.9) and DAPSA (48.0 vs 35.5) scores. Patients with enthesitis also had a higher HAQ-DI (1.4 vs 1.1) and a lower mean SF-36 PCS (32.2 vs 35.5) score (Table 1), indicative of worse physical functioning.

### Dactylitis outcomes

**Figure 1.**
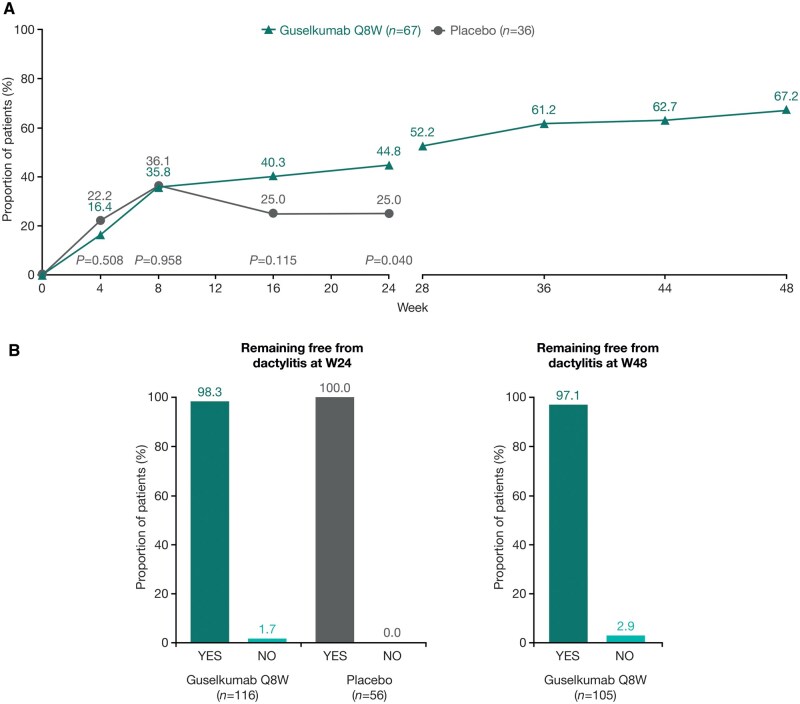
Proportion of patients with dactylitis resolution over time, and remaining free from dactylitis at W24 and W48. Panel (**A**) shows the proportion of patients with dactylitis resolution over time, among those with DSS ≥ 1 at baseline. Panel (**B**) shows the proportion of patients remaining free from dactylitis at W24 and W48, among those with DSS = 0 at baseline. NRI/composite analysis used through W24; NRI analysis only beyond W24. DSS, Dactylitis Severity Score; NRI, non-responder imputation; Q8W, every 8 weeks; W, Week

Dactylitis resolution rates at W24 and W48 in patients with DSS ≥1 at baseline have been reported previously [23]. The rate of dactylitis resolution was numerically higher in the guselkumab group than in the placebo group at W16 (27/67 [40.3%] vs 9/36 [25.0%], respectively), reaching nominal significance at W24 (30/67 [44.8%] vs 9/36 [25.0%]; Fig. 1A). At W24, the number needed to treat (NNT) with guselkumab to achieve an additional dactylitis resolution event was 5.1 patients. In the continuous guselkumab group, response rates continued to improve through W48 (W28, 35/67 [52.2%]; W36, 41/67 [61.2%]; W44, 42/67 [62.7%] and W48, 45/67 [67.2%]).

Patients who did not have dactylitis at baseline generally remained dactylitis-free at W24 (guselkumab group, 114/116 [98.3%]; placebo group, 56/56 [100.0%]) and at W48 (patients randomized to guselkumab, 102/105 [97.1%]; Fig. 1B).

**Figure 2.**
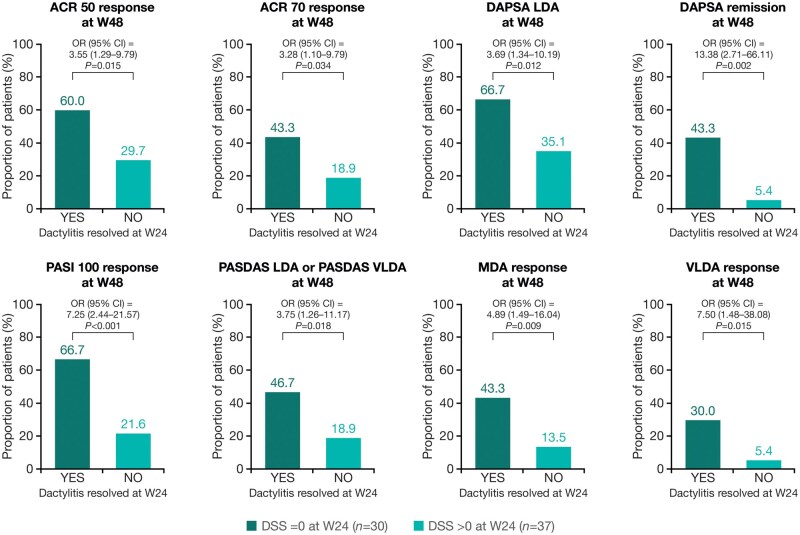
Associations between dactylitis resolution at W24 and achievement of stringent measures of disease control at W48. Patients analysed were those randomized to guselkumab with DSS ≥ 1 at baseline. ACR, American College of Rheumatology; CI, confidence interval; DAPSA, Disease Activity in Psoriatic Arthritis; DSS, Dactylitis Severity Score; LDA, low disease activity; MDA, minimal disease activity; OR, odds ratio; PASDAS, Psoriatic Arthritis Disease Activity Score; PASI, Psoriasis Area and Severity Index; VLDA, very low disease activity; W, Week

Patients treated with guselkumab who achieved dactylitis resolution at W24 were more likely (*P* < 0.05), compared with those who did not achieve dactylitis resolution, to achieve disease control at W48 assessed using stringent joint (ACR 50: OR 3.55 [95% CI 1.29–9.79]; ACR 70: 3.28 [1.10–9.79]; DAPSA LDA: 3.69 [1.34–10.19]; DAPSA remission: 13.38 [2.71–66.11]), skin (PASI 100: 7.25 [2.44–21.57]) and composite measures (PASDAS LDA or PASDAS VLDA: 3.75 [1.26–11.17]; MDA: 4.89 [1.49–16.04] and VLDA: 7.50 [1.48–38.08]; Fig. 2).

### Enthesitis outcomes

**Figure 3.**
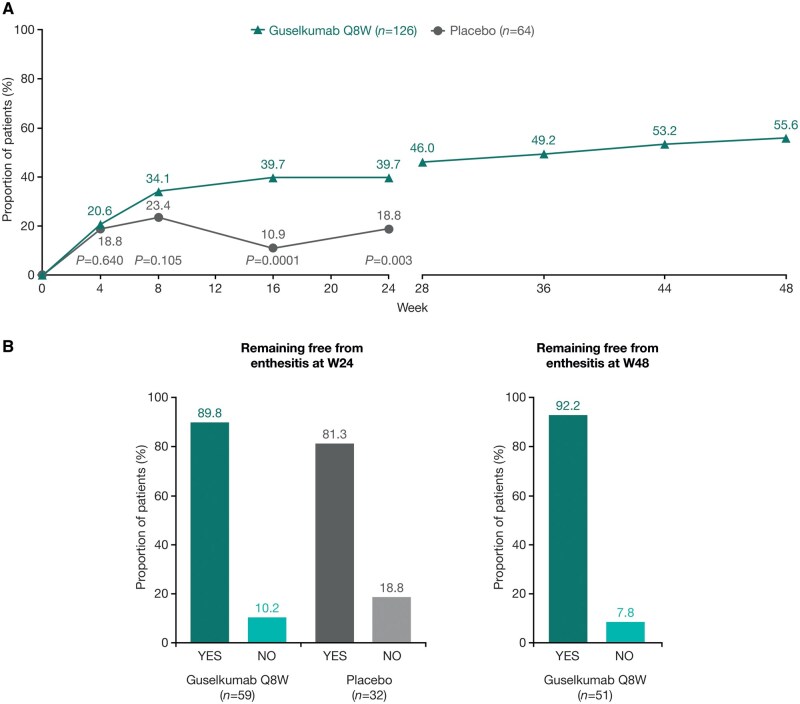
Proportions of patients with enthesitis resolution over time, and remaining free from enthesitis at W24 and W48. Panel (**A**) shows the proportion of patients with enthesitis resolution over time, among those with LEI ≥ 1 at baseline. Panel (**B**) shows the proportion of patients remaining free from enthesitis at W24 and W48, among those with LEI = 0 at baseline. NRI/composite analysis used through W24; NRI analysis only beyond W24. LEI, Leeds Enthesitis Index; NRI, non-responder imputation; Q8W, every 8 weeks; W, Week

Enthesitis resolution rates at W24 and W48 in patients with LEI ≥1 at baseline have been reported previously [23]. The rate of enthesitis resolution was numerically higher in the guselkumab group vs the placebo group at W8 (43/126 [34.1%] vs 15/64 [23.4%]), reaching nominal significance at W16 (50/126 [39.7%] vs 7/64 [10.9%]) and W24 (50/126 [39.7%] vs 12/64 [18.8%]; Fig. 3A). At W24, the NNT with guselkumab for one more patient to have resolution of enthesitis was 4.8 patients. In the continuous guselkumab group, response rates continued to improve through to W48 (W28, 58/126 [46.0%]; W36, 62/126 [49.2%]; W44, 67/126 [53.2%] and W48, 70/126 [55.6%]).

A high proportion of the patients who did not have enthesitis at baseline remained enthesitis free at W24 (guselkumab group, 53/59 [89.8%]; placebo group, 26/32 [81.3%]) and at W48 (patients randomized to guselkumab, 47/51 [92.2%]; Fig. 3B).

**Figure 4.**
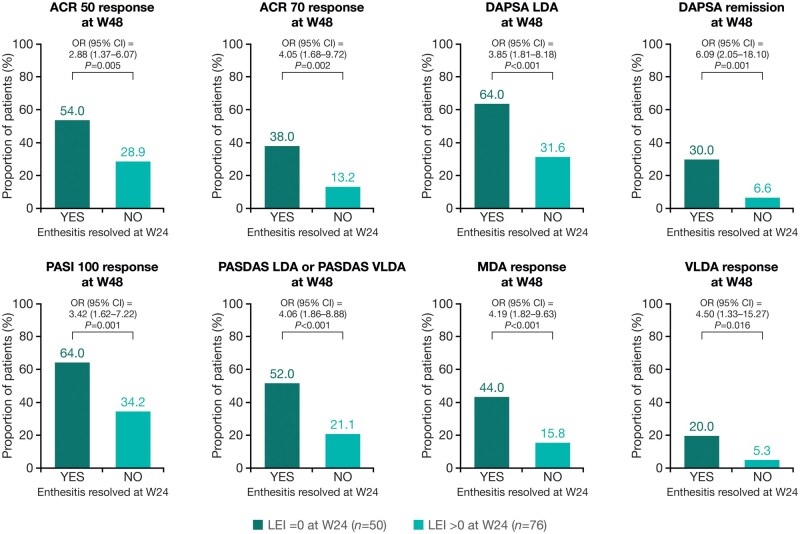
Associations between enthesitis resolution at W24 and achievement of stringent measures of disease control at W48. Patients analysed were those randomized to guselkumab with LEI ≥ 1 at baseline. ACR, American College of Rheumatology; CI, confidence interval; DAPSA, Disease Activity in Psoriatic Arthritis; LDA, low disease activity; LEI, Leeds Enthesitis Index; MDA, minimal disease activity; OR, odds ratio; PASDAS, Psoriatic Arthritis Disease Activity Score; PASI, Psoriasis Area and Severity Index; VLDA, very low disease activity; W, Week

As with those who achieved dactylitis resolution, patients treated with guselkumab who achieved enthesitis resolution at W24 were more likely (*P* < 0.05) to achieve disease control at W48 defined using stringent joint (ACR 50: OR 2.88 [95% CI 1.37–6.07]; ACR 70: 4.05 [1.68–9.72]; DAPSA LDA: 3.85 [1.81–8.18]; DAPSA remission: 6.09 [2.05–18.10]), skin (PASI 100: 3.42 [1.62–7.22]) and composite measures (PASDAS LDA or PASDAS VLDA: 4.06 [1.86–8.88]; MDA: 4.19 [1.82–9.63] and VLDA: 4.50 [1.33–15.27]), compared with patients whose enthesitis was not resolved (Fig. 4).

## Discussion

Current treatment recommendations advocate the use of biologic therapy in patients with active enthesitis or dactylitis who have insufficient response to NSAIDs or local steroids, irrespective of any prior use of csDMARDs [30, 31]. In these *post hoc* analyses of the COSMOS trial, patients with active TNFi-IR PsA and dactylitis and/or enthesitis who were randomized to guselkumab treatment had higher dactylitis and enthesitis resolution rates than patients randomized to placebo. Approximately half of patients randomized to placebo used MTX at baseline and were permitted to continue use during the study, which may have contributed to the moderate rates of dactylitis and enthesitis resolution seen in the placebo group through W24. Notably, in COSMOS, among those randomized to guselkumab the increase in resolution rates from W24 to W48 was more pronounced than in a pooled analysis of DISCOVER-1 and DISCOVER-2 (which enrolled predominately biologic-naïve patients) for dactylitis (22.4% vs 16.2%) and enthesitis (15.9% vs 8.2%) [4, 10]. Therefore, even in a TNFi-IR patient population, guselkumab provided a consistent increase in dactylitis and enthesitis resolution response rates over time, with no plateau in response observed.

Overall, the rates of resolution are generally consistent with those observed in other clinical trials of therapies targeting dactylitis and enthesitis pathogenesis (including IL-17A, TNF, Janus kinase inhibitors and other IL-23 inhibitors) [32–37]. However, precise comparisons are limited owing to differences in study designs. Also consistent with previous findings [4, 10], we showed that during guselkumab treatment over a period of 48 weeks, new-onset dactylitis or enthesitis was uncommon. Further studies are required to assess whether guselkumab has a preventative role in the development of these disease domains.

Importantly, patients treated with guselkumab who achieved dactylitis or enthesitis resolution at W24 were more likely to achieve stringent measures of disease control at W48, including joint (ACR 50/70; DAPSA low/remission), skin (PASI 100) and multi-domain composite (PASDAS LDA or PASDAS VLDA; MDA; VLDA) outcomes than those who did not. These findings are consistent with other reports, including the pooled DISCOVER-1 and DISCOVER-2 analysis, detailing the positive association between dactylitis and enthesitis resolution and achievement of disease control endpoints [4, 10, 32]. Additionally, resolution of these periarticular manifestations has been linked to improvements in patient-reported outcomes, including fatigue, pain, physical functioning and health-related quality of life [5, 32]. This is particularly relevant for TNFi-IR PsA patients, who often experience worse symptoms than biologic-naïve patients [21]. Therefore, identifying and treating dactylitis and enthesitis may not only alleviate disease burden but also improve overall patient quality of life and long-term prognosis.

Patients in COSMOS with baseline dactylitis exhibited higher mean SJC, DAPSA, PASI, psoriatic BSA and PGA scores than those without dactylitis, aligning with evidence that dactylitis in PsA is indicative of more severe disease across multiple disease domains [4, 6, 8, 10, 11, 32]. Similarly, at baseline, patients with enthesitis, compared with those without, had higher mean SJC, TJC and DAPSA scores, reflecting more severe joint involvement. These patients also had higher HAQ-DI (representing greater disability) and lower SF-36 PCS (representing worse bodily function) scores. These findings highlight the associations between periarticular manifestations and overall disease burden, particularly in joint involvement and physical functioning. These disease activity scores in patients with enthesitis are similar to those observed in a cluster of patients with PsA characterized by enthesitis and large joint involvement, which was identified via machine learning from DISCOVER-1 and DISCOVER-2 data [29]. These findings may aid in identifying patients with these periarticular manifestations. As expected, our data showed that patients with dactylitis were more likely to also have enthesitis than those without dactylitis, and *vice versa*, underscoring the associated nature of these two conditions [9].

A limitation of our *post hoc* analyses is that they were not powered for statistical significance, owing to their exploratory nature. Moreover, the evaluation of dactylitis and enthesitis was conducted using the DSS and the LEI, respectively, instead of utilizing imaging methods that can detect inflammatory changes. As a result, inter- and intra-observer reliability may vary [24]. Nonetheless, these *post hoc* analyses are strengthened by the large number of patients enrolled in COSMOS, all of whom had TNFi-IR PsA. This allowed for evaluation of the impact of dactylitis or enthesitis resolution on long-term disease outcomes in a population of patients typically considered to be less responsive to subsequent biologic therapies [21, 22]. Furthermore, these analyses employed stringent and validated outcome measures to assess disease control at W48.

In summary, these findings from COSMOS have clinically relevant implications for routine patient care. Dactylitis and enthesitis are associated with a greater disease burden and worse prognosis, highlighting the importance for physicians to identify these conditions and provide adequate treatment. The importance of treating these manifestations of PsA is emphasized by the fact that patients treated with guselkumab who achieved dactylitis or enthesitis resolution at W24 were more likely to achieve joint, skin and composite stringent measures of disease control at W48 compared with those who did not. These data, coupled with the key role of IL-23 in dactylitis and enthesitis pathogenesis [13–15], suggest guselkumab is a highly effective treatment option for resolving these disease domains. Even among patients who do not experience early resolution of these conditions, continued guselkumab treatment may be valuable since resolution rates increased through to W48 without plateauing.

## Supplementary Material

keaf490_Supplementary_Data

## Data Availability

The data sharing policy of Johnson & Johnson Innovative Medicine is available at https://www.janssen.com/clinical-trials/transparency. As noted on this site, requests for access to the study data can be submitted through Yale Open Data Access (YODA) Project site at http://yoda.yale.edu.

## References

[1] FitzGerald O , Ogdie A, Chandran V et al Psoriatic arthritis. Nat Rev Dis Primers 2021;7:59. 34385474 10.1038/s41572-021-00293-y

[2] Ritchlin CT , Colbert RA, Gladman DD. Psoriatic arthritis. N Engl J Med 2017;376:2095–6. 10.1056/NEJMc1704342 28538114

[3] Taylor W , Gladman D, Helliwell P et al; CASPAR Study Group. Classification criteria for psoriatic arthritis: development of new criteria from a large international study. Arthritis Rheum 2006;54:2665–73. 16871531 10.1002/art.21972

[4] McGonagle D , McInnes IB, Deodhar A et al Guselkumab, a selective interleukin-23 p19 subunit inhibitor, resolves dactylitis in patients with active psoriatic arthritis: pooled results through Week 52 from two Phase 3 studies. ACR Open Rheumatol 2023;5:227–40. 36880890 10.1002/acr2.11537 PMC10100698

[5] Rahman P , McInnes IB, Deodhar A et al Association between enthesitis/dactylitis resolution and patient-reported outcomes in guselkumab-treated patients with psoriatic arthritis. Clin Rheumatol 2024;43:1591–604. 38472528 10.1007/s10067-024-06921-8 PMC11018666

[6] Dubash S , Alabas OA, Michelena X et al Dactylitis is an indicator of a more severe phenotype independently associated with greater SJC, CRP, ultrasound synovitis and erosive damage in DMARD-naive early psoriatic arthritis. Ann Rheum Dis 2022;81:490–5. 34893470 10.1136/annrheumdis-2021-220964 PMC8921567

[7] Gladman DD , Ziouzina O, Thavaneswaran A, Chandran V. Dactylitis in psoriatic arthritis: prevalence and response to therapy in the biologic era. J Rheumatol 2013;40:1357–9. 23818708 10.3899/jrheum.130163

[8] Mease PJ , Karki C, Palmer JB et al Clinical characteristics, disease activity, and patient-reported outcomes in psoriatic arthritis patients with dactylitis or enthesitis: results from the Corrona Psoriatic Arthritis/Spondyloarthritis Registry. Arthritis Care Res (Hoboken) 2017;69:1692–9. 28376239 10.1002/acr.23249

[9] Tinazzi I , McGonagle D, Aydin SZ et al 'Deep Koebner’ phenomenon of the flexor tendon-associated accessory pulleys as a novel factor in tenosynovitis and dactylitis in psoriatic arthritis. Ann Rheum Dis 2018;77:922–5. 29511028 10.1136/annrheumdis-2017-212681

[10] McGonagle D , McInnes IB, Deodhar A et al Resolution of enthesitis by guselkumab and relationships to disease burden: 1-year results of two phase 3 psoriatic arthritis studies. Rheumatology (Oxford) 2021;60:5337–50. 33822898 10.1093/rheumatology/keab285 PMC8566200

[11] Polachek A , Li S, Chandran V, Gladman DD. Clinical enthesitis in a prospective longitudinal psoriatic arthritis cohort: incidence, prevalence, characteristics, and outcome. Arthritis Care Res (Hoboken) 2017;69:1685–91. 27998023 10.1002/acr.23174

[12] Bridgewood C , Watad A, Russell T et al Identification of myeloid cells in the human enthesis as the main source of local IL-23 production. Ann Rheum Dis 2019;78:929–33. 31018959 10.1136/annrheumdis-2018-214944 PMC6585277

[13] Bianchi E , Rogge L. The IL-23/IL-17 pathway in human chronic inflammatory diseases—new insight from genetics and targeted therapies. Microbes Infect 2019;21:246–53. 31252215 10.1016/j.micinf.2019.06.009

[14] Vecellio M , Hake VX, Davidson C et al The IL-17/IL-23 axis and its genetic contribution to psoriatic arthritis. Front Immunol 2020;11:596086. 33574815 10.3389/fimmu.2020.596086 PMC7871349

[15] Ray K. Inflammation: IL-23 tees off enthesitis. Nat Rev Immunol 2012;12:552–3. 10.1038/nri3269 22828907

[16] Boehncke WH , Brembilla NC, Nissen MJ. Guselkumab: the first selective IL-23 inhibitor for active psoriatic arthritis in adults. Expert Rev Clin Immunol 2021;17:5–13. 33251833 10.1080/1744666X.2020.1857733

[17] Johnson & Johnson. Tremfya^®^ (guselkumab) approved by US Food and Drug Administration as the first selective interleukin (IL)-23 inhibitor for active psoriatic arthritis. 2020. https://www.jnj.com/media-center/press-releases/tremfya-guselkumab-approved-by-u-s-food-and-drug-administration-as-the-first-selective-interleukin-il-23-inhibitor-for-active-psoriatic-arthritis (11 November 2024, date last accessed).

[18] Johnson & Johnson. Tremfya^®^ (guselkumab) receives U.S. FDA approval for adults with moderately to severely active ulcerative colitis, strengthening Johnson & Johnson’s leadership in inflammatory bowel disease. 2024. https://www.jnj.com/media-center/press-releases/tremfya-guselkumab-receives-u-s-fda-approval-for-adults-with-moderately-to-severely-active-ulcerative-colitis-strengthening-johnson-johnsons-leadership-in-inflammatory-bowel-disease (31 December 2024, date last accessed).

[19] Deodhar A , Helliwell PS, Boehncke W-H et al; DISCOVER-1 Study Group. Guselkumab in patients with active psoriatic arthritis who were biologic-naive or had previously received TNFalpha inhibitor treatment (DISCOVER-1): a double-blind, randomised, placebo-controlled phase 3 trial. Lancet 2020;395:1115–25. 32178765 10.1016/S0140-6736(20)30265-8

[20] Mease PJ , Rahman P, Gottlieb AB et al; DISCOVER-2 Study Group. Guselkumab in biologic-naive patients with active psoriatic arthritis (DISCOVER-2): a double-blind, randomised, placebo-controlled phase 3 trial. Lancet 2020;395:1126–36. 32178766 10.1016/S0140-6736(20)30263-4

[21] Glintborg B , Ostergaard M, Krogh NS et al Clinical response, drug survival, and predictors thereof among 548 patients with psoriatic arthritis who switched tumor necrosis factor alpha inhibitor therapy: results from the Danish Nationwide DANBIO Registry. Arthritis Rheum 2013;65:1213–23. 23460467 10.1002/art.37876

[22] Harrold LR , Stolshek BS, Rebello S et al Impact of prior biologic use on persistence of treatment in patients with psoriatic arthritis enrolled in the US Corrona registry. Clin Rheumatol 2017;36:895–901. 28271234 10.1007/s10067-017-3593-x

[23] Coates LC , Gossec L, Theander E et al Efficacy and safety of guselkumab in patients with active psoriatic arthritis who are inadequate responders to tumour necrosis factor inhibitors: results through one year of a phase IIIb, randomised, controlled study (COSMOS). Ann Rheum Dis 2022;81:359–69. 34819273 10.1136/annrheumdis-2021-220991 PMC8862038

[24] Helliwell PS , Firth J, Ibrahim GH et al Development of an assessment tool for dactylitis in patients with psoriatic arthritis. J Rheumatol 2005;32:1745–50. 16142872

[25] Healy PJ , Helliwell PS. Measuring clinical enthesitis in psoriatic arthritis: assessment of existing measures and development of an instrument specific to psoriatic arthritis. Arthritis Rheum 2008;59:686–91. 18438903 10.1002/art.23568

[26] Felson DT , Anderson JJ, Boers M et al The American College of Rheumatology preliminary core set of disease activity measures for rheumatoid arthritis clinical trials. The Committee on Outcome Measures in Rheumatoid Arthritis Clinical Trials. Arthritis Rheum 1993;36:729–40. 8507213 10.1002/art.1780360601

[27] Helliwell PS , Deodhar A, Gottlieb AB et al Composite measures of disease activity in psoriatic arthritis: comparative instrument performance based on the efficacy of guselkumab in an interventional Phase II trial. Arthritis Care Res (Hoboken) 2020;72:1579–88. 31421033 10.1002/acr.24046 PMC7702129

[28] Fredriksson T , Pettersson U. Severe psoriasis—oral therapy with a new retinoid. Dermatologica 1978;157:238–44. 357213 10.1159/000250839

[29] Coates LC , Fransen J, Helliwell PS. Defining minimal disease activity in psoriatic arthritis: a proposed objective target for treatment. Ann Rheum Dis 2010;69:48–53. 19147615 10.1136/ard.2008.102053

[30] Coates LC , Soriano ER, Corp N et al; GRAPPA Treatment Recommendations domain subcommittees. Group for Research and Assessment of Psoriasis and Psoriatic Arthritis (GRAPPA): updated treatment recommendations for psoriatic arthritis 2021. Nat Rev Rheumatol 2022;18:465–79. 35761070 10.1038/s41584-022-00798-0 PMC9244095

[31] Gossec L , Kerschbaumer A, Ferreira RJO et al EULAR recommendations for the management of psoriatic arthritis with pharmacological therapies: 2023 update. Ann Rheum Dis 2024;83:706–19. 38499325 10.1136/ard-2024-225531 PMC11103320

[32] Kwatra SG , Khattri S, Amin AZ et al Enthesitis and dactylitis resolution with risankizumab for active psoriatic arthritis: integrated analysis of the randomized KEEPsAKE 1 and 2 trials. Dermatol Ther (Heidelb) 2024;14:1517–30. 38739215 10.1007/s13555-024-01174-4 PMC11169338

[33] Giles JT , Ogdie A, Gomez Reino JJ et al Impact of baseline body mass index on the efficacy and safety of tofacitinib in patients with psoriatic arthritis. RMD Open 2021;7:e001486. 33452181 10.1136/rmdopen-2020-001486 PMC7813423

[34] Mourad A , Gniadecki R. Treatment of dactylitis and enthesitis in psoriatic arthritis with biologic agents: a systematic review and metaanalysis. J Rheumatol 2020;47:59–65. 30824641 10.3899/jrheum.180797

[35] Orbai A-M , McInnes IB, Coates LC et al Effect of secukinumab on the different GRAPPA-OMERACT core domains in psoriatic arthritis: a pooled analysis of 2049 patients. J Rheumatol 2020;47:854–64. 31615919 10.3899/jrheum.190507

[36] Simons N , Degboe Y, Barnetche T et al Biological DMARD efficacy in psoriatic arthritis: a systematic literature review and meta-analysis on articular, enthesitis, dactylitis, skin and functional outcomes. Clin Exp Rheumatol 2020;38:508–15. 31969228

[37] Smolen JS , Mease P, Tahir H et al Multicentre, randomised, open-label, parallel-group study evaluating the efficacy and safety of ixekizumab versus adalimumab in patients with psoriatic arthritis naive to biological disease-modifying antirheumatic drug: final results by week 52. Ann Rheum Dis 2020;79:1310–9. 32660977 10.1136/annrheumdis-2020-217372 PMC7509529

