# Supplementary material for: Impact of dactylitis and enthesitis resolution on disease control in guselkumab-treated psoriatic arthritis patients with TNFi-IR: COSMOS post hoc analysis
Source: Rheumatology (Oxford). 2025 Sep 14;65(1):keaf490. doi: 10.1093/rheumatology/keaf490 (PMC12862382; doi:10.1093/rheumatology/keaf490)
Supplement: keaf490_Supplementary_Data [file keaf490_supplementary_data.pdf]

Supplementary material

Impact of dactylitis and enthesitis resolution on disease control in guselkumab-treated PsA patients with TNFi-IR: COSMOS *post-hoc* analysis

Helena Marzo-Ortega, Iain B. McInnes, Mohamed Sharaf, Alen Zabotti, Emmanouil Rampakakis, Dennis McGonagle, Ahmed Abogamal, Pascal Richette, Georg Schett

Supplementary Table S1. Baseline demographics and disease characteristics for patients randomized to guselkumab versus placebo (DSS ≥1 or LEI ≥1 at baseline)

Characteristic/measure	Dactylitis at baseline		Enthesitis at baseline	
	**Guselkumab (*n*=67)**	**Placebo (*n*=36)**	**Guselkumab (*n*=126)**	**Placebo (*n*=64)**
**Demographics**				
Age, years	49.4 (12.2)	46.3 (11.0)	50.0 (11.7)	49.6 (11.9)
<65 years, *n* (%)	58 (86.6)	35 (97.2)	112 (88.9)	59 (92.2)
≥65 years, *n* (%)	9 (13.4)	1 (2.8)	14 (11.1)	5 (7.8)
Sex				
Male, *n* (%)	30 (44.8)	21 (58.3)	51 (40.5)	31 (48.4)
Female, *n* (%)	37 (55.2)	15 (41.7)	75 (59.5)	33 (51.6)
Weight, kg	82.9 (18.3)	85.9 (23.2)	82.8 (18.0)[a]	93.3 (24.1)[a]
BMI, kg/m^2	28.6 (5.7)	28.3 (6.2)	28.9 (5.9)[a]	31.5 (7.1)[a]
**Joint measures**				
SJC, 0–66	12.9 (8.7)	11.1 (6.9)	11.4 (7.7)	9.6 (6.2)
TJC, 0–68	23.6 (14.0)	18.9 (10.0)	24.4 (13.8)	20.7 (11.5)
DAPSA score	52.0 (22.8)[a,b]	42.7 (15.9)[a]	50.2 (21.1)[a]	43.5 (16.6)[a]
**Periarticular manifestations**				
DSS ≥1, *n* (%)	67 (100.0)	36 (100.0)	52 (41.3)	26 (40.6)
DSS score, 1–60	6.7 (6.5)	7.4 (8.3)	7.4 (7.1)	8.8 (9.4)
LEI ≥1, *n* (%)	52 (77.6)	26 (72.2)	126 (100.0)	64 (100.0)
LEI score, 1–6	3.2 (1.4)	2.7 (1.4)	2.9 (1.5)	2.7 (1.5)
**Skin measures**				
PASI, 0–72	15.8 (14.0)[b]	12.4 (9.6)	12.4 (13.5)[c]	9.3 (9.4)
Psoriatic BSA, %	25.5 (25.5)	19.6 (19.4)	19.0 (23.8)	13.5 (19.2)

IGA <2, *n* (%)	11 (16.4)	9 (25.0)	32 (25.4)	16 (25.0)
IGA ≥2, *n* (%)	56 (83.6)	27 (75.0)	94 (74.6)	48 (75.0)
**Patient-reported measures**				
Patient pain, 0–10 cm VAS	7.0 (1.6)[a]	5.9 (1.6)[a]	6.6 (1.8)	6.1 (1.7)
HAQ-DI, 0–3	1.4 (0.5)[a]	1.1 (0.6)[a]	1.4 (0.6)	1.3 (0.6)
PtGA, 0–10 cm VAS	7.0 (1.6)[a]	6.2 (1.8)[a]	6.9 (1.8)	6.4 (1.6)
FACIT-F score, 0–52	30.8 (11.7)	30.3 (10.1)	28.6 (11.8)	28.6 (10.6)
SF-36 PCS score	32.9 (6.7)[a]	36.4 (6.5)[a]	31.9 (6.9)	33.0 (7.8)
SF-36 MCS score	48.3 (11.9)	45.8 (12.5)	47.7 (12.7)	46.8 (11.6)
**Physician-reported measures**				
PGA, 0–10 cm VAS	7.2 (1.2)[a]	6.5 (1.7)[a]	7.0 (1.4)[a]	6.5 (1.7)[a]
**CRP**, mg/dL	1.4 (2.1)[b]	0.9 (2.6)	1.3 (2.1)	0.8 (2.1)
**Prior/ongoing therapies**				
One prior TNFi, *n* (%)	60 (89.6)	31 (86.1)	111 (88.1)	56 (87.5)
Two prior TNFi, *n* (%)	7 (10.4)	5 (13.9)	15 (11.9)	8 (12.5)
Reason for prior TNFi discontinuation				
Efficacy, *n* (%)	54 (80.6)	29 (80.6)	102 (81.0)	53 (82.8)
Safety, *n* (%)	9 (13.4)	4 (11.1)	18 (14.3)	9 (14.1)
Other, *n* (%)	4 (6.0)	3 (8.3)	6 (4.8)	2 (3.1)
MTX use at baseline, *n* (%)	39 (58.2)	18 (50.0)	68 (54.0)	34 (53.1)

Data are mean (SD) unless otherwise stated.

[a]Guselkumab versus placebo $P<0.05$; [b]*n*=66; [c]*n*=125.

BMI, body mass index; BSA, body surface area; CRP, C-reactive protein; DAPSA, Disease Activity in Psoriatic Arthritis; DSS, Dactylitis Severity Score; FACIT-F, Functional Assessment of Chronic Illness Therapy – Fatigue; HAQ-DI, Heath Assessment Questionnaire – Disability Index; IGA, Investigator's Global Assessment; LEI, Leeds Enthesitis Index; MTX, methotrexate; PASI, Psoriasis Area and Severity Index; PGA, Physician's Global Assessment; PtGA, Patient's Global Assessment of arthritis; SD, standard deviation; SF-36 MCS, 36-item short-form health survey mental component summary; SF-36 PCS, 36-item short-form health survey physical component summary; SJC, swollen joint count; TJC, tender joint count; TNFi, tumour necrosis factor inhibitor; VAS, visual analogue scale.
